# Virtual Follow up After Distal Radius Fracture Surgery—Patient Experiences During the COVID-19 Pandemic

**DOI:** 10.1177/23743735231188819

**Published:** 2023-07-24

**Authors:** Linnea Arvidsson, Benjamin Hägglund, Lena Petersson, Eva Arvidsson, Magnus Tägil

**Affiliations:** 1 Department of Orthopaedics, Clinical Sciences, Lund University and Skåne University Hospital, Lund, Sweden; 2 166447 School of Health and Welfare, Halmstad University, Halmstad, Sweden; 3 Futurum, Jönköping, Sweden; 4 145651 School of Health and Welfare, Jönköping University, Jönköping, Sweden; 5 Department of Hand Surgery, Skåne University Hospital, Malmö, Sweden

**Keywords:** access to care, COVID-19, health literacy, patient satisfaction, qualitative methods, telemedicine, orthopedics, surgery

## Abstract

The majority of patients with a distal radius fracture (DRF) are elderly, a group known to experience difficulties with new technology, partly due to a low level of digital literacy. At the beginning of the coronavirus disease 2019 pandemic, during the spring 2020, patients that underwent DRF surgery had regular follow-ups replaced by video calls from their surgeon and physiotherapist. Afterward, patients answered questionnaires regarding health and digital literacy and took part in semistructured interviews regarding the experience of the virtual follow-up. By systemic text condensation, 2 major categories were identified: (1) The video call—new, but surprisingly simple: All but 1 found it easier than expected, and (2) Video calls—the patient's choice: All but 1 patient preferred video calls to physical visits for follow-up. This is the first mixed methods study to assess patients’ experiences of digital follow-up after DRF surgery. This study indicates that digital follow-up was highly appreciated, even among patients with low levels of digital literacy. Digital technologies must be made suitable even for patients with inadequate levels of digital literacy.

## Introduction

Recent studies have indicated that telemedicine systems appear to be safe for postoperative visits, saving time and money, and resulting in high satisfaction among patients and surgeons.^1–3^ Younger patients are more confident in using digital health tools.^4,^^5^ The distal radius fracture (DRF) is the most common fracture ^6^, and phone applications used to assist with rehabilitation have been studied.^7,^^8^ Patients who suffer a DRF are of mixed ages, but the majority are elderly, a group known to experience difficulties with new technology.^9^ The elderly as a group may, on the other hand, benefit substantially from telemedicine, something that became obvious during the pandemic.

To study the use of telemedicine by the elderly, the concept of functional digital literacy (FDL) has been introduced, similar to functional health literacy (FHL). FHL is a health literacy concept that covers basic skills in understanding and using health information.^10^ FDL, in a similar way, measures the ability to engage practically with new technology and requires operational, social, and creative skills.^11,^^12^

Studies of health literacy and digital literacy in the clinical setting are scarce.^12^ To the best of our knowledge, no mixed method studies, which use both interviews and surveys, have been performed to investigate the patient's experience of telemedicine after a surgically treated wrist fracture. Furthermore, knowledge about the feasibility of telemedicine, eg, video meetings for patients with DRFs, is lacking. Since the patients are expected to take part and be engaged in their own rehabilitation, health instructions need to be accessible and easy to understand.^11^ Thus, both FDL and FHL are important tools to study telemedicine, particularly in patients undergoing surgery.

Our aim was to study how patients with a DRF experienced video-based follow-up. We aimed to identify advantages as well as challenges with new digital technology. Since the approach included both digital technology and active participation by patients, we measured FDL and FHL in a mixed methods study.

## Method

In spring 2020, the coronavirus pandemic was escalating in our neighboring countries, and we prepared for a similar situation. We decided to follow all surgically-treated wrist fracture patients remotely, by video calls instead of face-to-face meetings. The patients received a video call via a secure link from the surgeon the day after surgery and from the physiotherapist 5 to 7 days after surgery. The video call started automatically when the patient pressed the link in a text message

### Participant Recruitment

At the Skane University Hospital in Lund, Sweden, about 250 patients with a DRF are operated on annually. During the study period of April and May 2020, only 12 patients underwent surgery at our hospital, due to limited surgical resources during the pandemic. For the study, the primary researcher invited all 12 patients during the hospital visit. Once verbal consent was provided, a time was arranged. To reduce the risk of having a homogeneous sample, there were no predetermined exclusion criteria.

### The Interviews

An interview guide was developed by the research team. The interview questions were concerned about (1) the experience of video calls as follow-up, and (2) the patients’ view on an “optimal follow-up.” The questions were open-ended and designed to allow the patients to provide their own experiences.

Semistructured interviews, 1 per patient, were conducted by telephone 6 to 8 weeks after surgery at the end of May and the beginning of June 2020 by an orthopedic resident (LA) with experience in the treatment of DRFs. The resident did not treat the participating patients. The interviews were 30 min long on average. All patients were at home during the interviews. The interviews were held in Swedish (the native language of the researchers and most of the patients) and are translated into English in this article. The interviews were transcribed verbatim.

### Analysis of the Interviews

The analytical steps were guided by systematic text condensation ^13^, a method that is common in medical qualitative research in Scandinavia because it is transparent and has a methodological approach. Three authors participated in the analysis: 1 specialist in orthopedic and hand surgery (MT), 1 resident in orthopedics (LA), and 1 physiotherapist specializing in rehabilitation after wrist trauma (BH). All read the transcripts individually, thoroughly, and repeatedly to get a sense of the full content. Each researcher listed their preliminary thoughts and themes using an inductive analytic approach eg, deriving primary themes directly from the raw data.^13,^^14^

**Figure 1.**
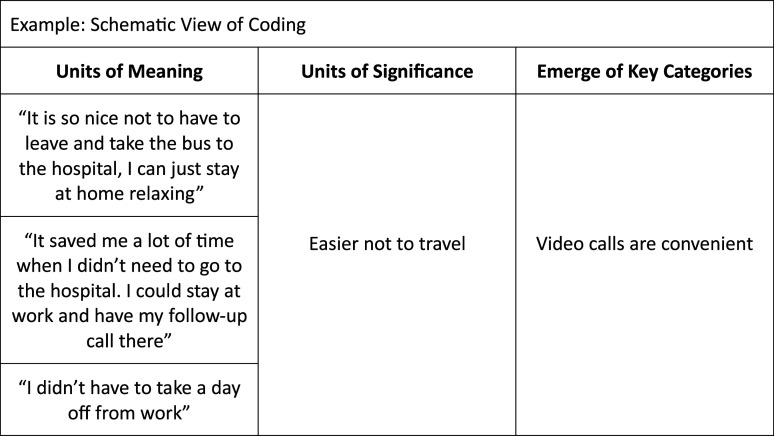
Schematic view of coding.

Discussions were held on how to interpret the content and the meaning of the preliminary themes. In the next step, the authors independently condensed the text, line-by-line, into meaning units, and labeled the units with a code. Subsequently, codes with similar content were sorted into code groups developed from the preliminary themes. The researchers’ meaning units, codes, and code groups were then compared and discussed within the whole research group, until consensus on relevant coding and code groups was reached (see examples of coding in Figure 1).

Next, the code groups were interpreted by the authors and developed into descriptions and concepts, and labeled with category headings. The categories were discussed between the researchers to ensure the consistency of the findings (triangulation). Finally, all 4 of the authors agreed upon the 2 “main categories” in the results.

### Questionnaire Data

In connection with the interview, all participants answered questionnaires regarding FHL and FDL.

**Figure 2.**
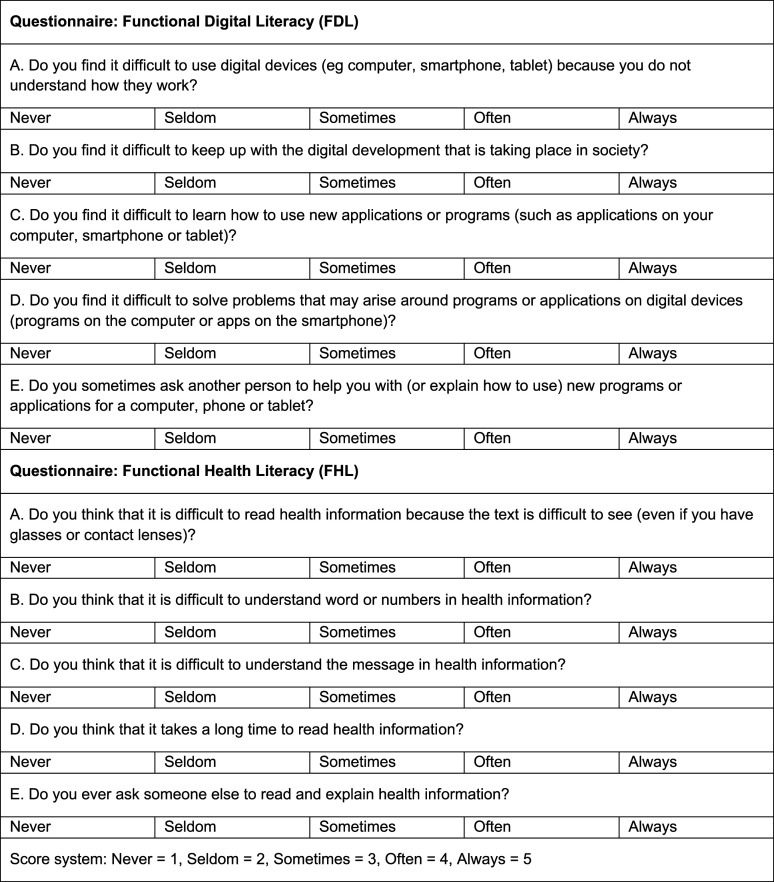
Functional digital literacy and health literacy questionnaires.

The Swedish FHL scale was used (Figure 2). The scale's first item focused on visual ability, related to the design of text and its accessibility. The following 2 items focused on the understanding of words and concepts, the fourth on perseverance in reading, and the fifth on the need for help in reading and understanding information. Responses were on a 5-point ordinal scale (1 = never to 5 = always), with lower scores indicating lower health literacy.^15^

To the best of our knowledge, there is no equivalent questionnaire to measure the patient's FDL. Hence, a questionnaire measuring patients’ confidence levels and their attitudes toward digital technology was developed (Figure 2). The first question focused on general digital competence regarding the use of digital tools. The second question focused on the attitude to digital transformation in society. The third question focused on the ability to learn how to use new software, and the last 2 questions focused on the ability to solve problems concerning the software.

### Analysis of the Questionnaires

The Swedish FHL scale does not provide an initial summary score. Instead, reported variables are recategorized into 3 different levels (inadequate, problematic, or sufficient health literacy). Thus, FHL was categorized using the recommended transformations: 1 and 2 became 1; 3 became 100; and 4 and 5 became 1000. All values were summed. Scores exceeding 1000 were interpreted as an inadequate FHL, values between 100 and 1000 as problematic, and values below 100 as sufficient.^15^ The same analytics procedure was used for the FDL scale.

### Reporting

To ensure clear reporting of the study, we have followed the COREQ checklist for reporting qualitative research.^16^

## Results

Ten out of 12 invited patients participated in the study. One patient was unable to participate due to cognitive impairment, and 1 due to language issues. Of the participants, 3 were men and 7 were women, and the age ranged from 32 to 85 years. Two patients were in their thirties, 2 in their forties, 4 in their fifties, 1 in her 60s, and 1 patient was over 80 years old. Four patients did not have Swedish as their native language.

The questionnaires showed that a majority of the participants had sufficient levels of FHL. However, only 4 of the 10 patients had sufficient levels of FDL, 3 had inadequate levels, and 3 had problematic levels. The levels of FHL and FDL are presented in Table 1.

**Table 1.** Results: Functional Digital and Health Literacy Questionnaires.

*Results:* Questionnaire for self-reported digital literacy						
	Questions					
Participants	A	B	C	D	E	Score
P1	1	3	2	3	3	302
P2	1	1	1	1	1	5
P3	1	1	1	2	1	5
P4	1	1	2	2	2	5
P5	4	4	5	3	4	4100
P6	1	1	3	3	3	302
P7	1	3	1	4	3	1202
P8	3	1	3	1	4	1202
P9	1	1	1	1	1	5
P10	1	3	2	2	1	104
*Results:* Questionnaire for self-reported health literacy						
	*Questions*					
Participants	A	B	C	D	E	Score
P1	1	1	1	1	1	5
P2	1	3	3	1	1	203
P3	1	1	1	1	1	5
P4	1	1	2	1	2	5
P5	1	1	1	1	1	5
P6	1	1	1	1	1	5
P7	1	1	1	1	1	5
P8	1	1	1	1	1	5
P9	1	1	1	1	3*	104
P10	1	1	1	3	1	104

*The patient comments that since Swedish is not his first language, he sometimes has trouble understanding certain words.

In the interviews, the 2 main categories were identified: “The video call—new, but surprisingly simple” and “The video call—The patient's choice”.

### The Video Call—new, but Surprisingly Simple

The majority expressed no problems participating in a video call.

The video call was very easy. Usually you have to download something, but here it was, just press a link and it just worked! It was really good.—Participant 9

Some patients with inadequate FDL worried whether they would be able to cope with the technology. However, all but one of these found it easier than expected to connect.

At first I thought: I will never be able to do this. I thought it would be really hard. But it went just fine!—Participant 5

My husband was home, so I asked him to connect. It seemed easy, I’m 100% sure I could do it on my own if I had to, but since he was home I didn’t—Participant 8

The patients described that it was easy to show the physiotherapist and surgeon, through the video, how they performed their hand exercises.

The doctor sort of carefully checked the exercises I did and pushed me to do the finger exercises properly. It went very well.—Participant 2

The oldest patient mentioned that she received help to connect from her partner of the same age. She said she could not manage on her own.

Well, of course it is good if you know how to handle it. I could never connect and do all of it on my own. But I went to my partner, so we could do it on his phone. And he managed to connect. And then I thought it worked really well actually. But you must know how to handle it … I’m 85 years old! I wasn’t born with all these technical things.—Participant 7

Many patients emphasized the fact that the technical solution for the video call itself must be simple. Yet even simple technical solutions can be experienced as difficult, both for the patient and the clinician.

It was very easy since I just had to press a link in a text message. Sometimes you must install new programs and applications, I don’t like that.—Participant 2

The first call went technically fine. The discussion with the physiotherapist and the content of the meeting were very good, but we had some technical difficulties. It just went off. But to start it again just took a minute. I could just press the link again … so we had some issues, but since it was very easy to connect again, I wasn’t bothered. But for someone who is not technically oriented it would have been confusing.—Participant 9

### The Video Call—The Patient's Choice

All but 1 patient preferred video calls over physical visits as the first follow-up after surgery. The participants emphasized how comfortable and easy it was to not travel and not take time off from work.

I really liked the video call; it saves a lot, especially the travel to the hospital. Maybe it's a benefit that the doctor can also be anywhere. It provides freedom for both the doctor and the patient.—Participant 2

The video call is the best! I thought: Oh excellent, now I don’t need to go to the hospital again just to talk … to be able to sit in peace and quiet at home, it works just as well as to be in a room at the hospital.—Participant 6

For me, the video call is absolutely the best, I didn’t have to take a day off from work.—Participant 1

The patients also mentioned the advantages of environmental benefits of not traveling.

I would really like the follow-ups to be like this, with digital video calls. Because if we think about the environment and, depending on where you live, take into account the time it takes to travel to the hospital. It was a very positive experience.—Participant 3

One participant emphasized that even if she needed assistance, it was easier for her to get help with making the video call than to get help with transportation to the hospital and back.

I could not handle the technique. But I would not manage to go to the hospital by myself either; I would have to ask someone for help. People don’t have time to help me get to the hospital, but to help me with the phone is easy for someone who knows how to do it.—Participant 7

## Discussion

Earlier studies on telemedicine in orthopedics are diverse, using various methods and outcome measures, and the findings are sometimes difficult to interpret and generalize.^17^ In a systematic review, 41 studies on telemedicine in orthopedics were included, and a trend was shown toward high patient satisfaction using digital follow-up.^17^ There are, however, limitations to telemedicine. The physical proximity between doctor and the patient, emphasized as helping to create trust and reassurance,^18^ is absent in teleconsultations. Even if advanced virtual examination techniques are being developed,^19^ telemedicine does not allow ordinary physical examination of the patient, which is usually considered an essential component of an orthopedic assessment.^20^ In order to still benefit from the many unique advantages of digital care, a hybrid model of care can be used. For example, return visits can be carried out digitally. According to surgeons, the postoperative patient is the most suitable for digital follow-up, with a 79% surgeon satisfaction rate, whereas first-time visits are considered the least suitable.^3^

The first visit after wrist surgery mainly addresses the postoperative swelling and emphasizing correct hand exercises; for this, the digital video meeting is ideal. The patient's mobility can be visually examined and misunderstandings in technique easily corrected. In our study, the early rehabilitation result was visually checked once again by the physiotherapist, giving a second opportunity to intervene if the result was unsatisfactory.

By using mixed methods, we aimed to achieve a deeper understanding of patients’ experiences of digital follow-up. In 2020, the safety and efficacy of telemedicine were studied in general surgery ^1^ and the authors concluded that their telemedicine program was safe, timesaving, and had extremely high patient satisfaction. Interestingly, quotes from the patients were included, although they were anecdotal and not relevant to the study analysis and aim. To us, this indicates an interest in describing the patients’ true and deeper personal opinions and experiences of telemedicine rather than a focus on just measuring the results in scores and questionnaires.

We were able to show that the benefits of video meetings are many, but we also identified clear challenges that require careful consideration to enable the digital health transition. From the interviews, we learned that half of the study participants expressed a fear or some degree of worry beforehand of not being able to handle the technology. In general, the FHL level was high, whereas the FDL was lower. The patients expressed relief once the technology turned out to work. In hindsight, the patients (including the patients who scored low on the FDL scale) concluded that the technology was relatively simple to use.

In general, elderly people are considered more resistant to using new technologies, but maybe that is a myth. It has also been suggested that the elderly are underrepresented in studies of digital health research.^21^ In an emergency department-based, telemedicine program, the satisfaction scores among the older patients were similar to those of the younger patients.^22^ In Sweden, high Internet use is reported in general. Declining use is seen with increasing age, but still, 93% of people aged 62–71 use the internet daily, 83% in the age group 72–81, and 50% in the groups aged 82 or older.^4^ With a high number of nonusers in the super elderly, a challenge exists for the digital transition in healthcare, in particular in diagnoses such as DRF with patients ranging from the young to the super elderly.

The coronavirus pandemic served as a catalyst for the rapid development of tools to conduct digital care. The acute state of emergency, and the urge to reduce the risk of infection for patients and clinicians, made it possible to temporarily overlook several legal and security aspects when implementing telemedicine.^23,^^24^ Now that the situation is not as acute, in order to continue the use of digital care, there is an urgent need to develop rules of application to ensure quality, safety, liability, and confidentiality.^23^^–26^ The patients in our study emphasized the importance of easy-to-use technology, but this will become a balancing act when IT-security is of the highest priority.

## Limitations

Our study had limitations. Although all available patients were asked to participate, the study was small and single site, and the findings may be difficult to generalize to a larger patient group or another setting. All patients participating in the study accepted follow-up by video consultation. It would have added to the study to interview someone who was initially more skeptical or negative about telemedicine. Two patients declined to be interviewed due to cognitive impairment and/or language difficulties, which highlights the limitations of the follow-up method.

## Conclusions

In the transition into telemedicine, the needs and capabilities of heterogeneous groups of patients must be considered. The technology needs to be adapted to patients with inadequate or problematic FDL. In our study, the patients managed to handle the new technology, despite a low FDL. This study also indicates that digital follow-up after DRF surgery is highly appreciated even among patients with low FDL.

We conclude that the technology was well adapted for our audience. In the future, we hypothesize that digital follow-up will be the new standard, at least partly. In our society, the aim is to provide equal care. We, therefore, need to ensure that digital technologies are made suitable for people with inadequate levels of FDL. The patients are ready for the change, but healthcare needs to optimize the tools for digital meetings. Patients, as well as medical staff, deserve tools with intuitive design and high levels of usability.
